# Use of and factors associated with self-treatment in China

**DOI:** 10.1186/1471-2458-12-995

**Published:** 2012-11-17

**Authors:** Li Yuefeng, Rao Keqin, Ren Xiaowei

**Affiliations:** 1 Center for Health Statistics and Information, Ministry of Health, Xizhimen South Road, Beijing 100044, China; 2 School of Public Health, Lanzhou University, Donggangxi Road, Lanzhou, Gansu 730000, China

**Keywords:** Self-treatment decisions, Multinomial logit selection model, China national health survey

## Abstract

**Background:**

When an individual is ill or symptomatic, they have the options of seeking professional health care, self-treating or doing nothing. In China, some studies suggest that the number of individuals opting to self-treat has been rapidly increasing in recent years. Therefore, the aim of this study was to analyze the trends of and factors related to self-treatment in China.

**Methods:**

Self-treatment was measured based the concept and data of the China National Health Survey (CNHS), which covers 802,454 individuals. We used CNHS data from 1993, 1998, 2003, and 2008, and a Multinomial Logit Selection Model to estimate the factors influencing the decision to self-treat.

**Results:**

The prevalences of self-treatment with a recall period of two-weeks were significantly higher in urban compared with rural areas (31.2% vs 14.9% in 1993, 43.5% vs 21.4% in 1998, 47.2% vs 31.4% in 2003, 31.0% vs 25.3% in 2008) in China. Economic (per capita income, TV, sanitary water) and individual (education, profession, family members, exercise) factors, as well as accessibility to drugs had a positive association with the probability of self-treating. Different illness symptoms, severity, and duration show a negative association with the probability of self-treating, showing a degree of rationality in decision-making. Different insurance systems were also found to have an effect on self-treatment decision-making.

**Conclusions:**

Self-treatment and professional medical services have shared the incremental medical needs of residents in recent years in China. Self-perceived illness status, economic circumstances, and education play important roles in health care decision-making.

## Background

When ill, individuals can seek professional health care, self-treat or do nothing [1]. Self-treatment has existed long before professional health care. An estimated 70%–95% of all illnesses are managed without the intervention of a physician [2-5]. The fact that the majority of symptoms and complaints are self-treated has been described using the metaphor "iceberg of symptoms" [6,7].

In many developing countries, self-medication is common practice because of concerns related to the quality of health care delivery systems and skepticism about the benefits of professional health care vis-a-vis traditional medicine [8]. Although many Chinese people have a strong attachment to traditional home remedies, most people still use drugs, therefore self-treatment mainly includes self-medication [9].

Medicines for self-treatment are often called ‘over the counter’ (OTC) drugs and are available without a doctor's prescription through pharmacies. A switch of prescription drugs to over the counter (OTC) drugs has been implemented in China since 1999 [10]. China has successively publicized about 5000 types of OTC drugs, including Chinese patent medicines.

Self-treatment is a rapidly increasing trend in China [11]. Previous studies have indicated that individuals resolve uncomfortable symptoms, influenza and skin discomforts through self-treatment. More than 80% of the respondents of one study considered that they would carry out self-treatment through purchasing OTC drugs [12,13]. There are several potential reasons for the rapidly increasing use of self-treatment in China. First, the increase may be partly explained by the rapid inflation in the price of professional medical services. For example, per capita health expenditure increased from 116.3 RMB in 1993 to 1062 RMB in 2008, and its percentage of GDP increased from 3.90% in 1993 to 4.69% in 2008 [14]. Some urban and many rural populations that cannot afford such medical expenditure have to use lower cost drugs to treat their illnesses [15]. Second, population aging and more complex caseloads may explain the rapid increase in self-treatment witnessed in recent years [16]. The proportion of persons older than 60 years is increasing by 3.2% per year in China. In many cases these elderly people rely on self-treatment because of the economic and time costs involved in seeking professional health care [17]. Third, in rural areas, long distances from health facilities and poor quality of health services at community health centers have been cited as possible reasons for the continued growth of self-treatment [9,17]. Fourth, the credibility of medical institutes is being damaged because of their excessive and wasteful use of drugs and examinations; some studies found that about 20%–30% of medical resources are wasted [18]. Some patients who are able to pay have given up medical services for self-medication and non-medical consumption [19-21]. Fifth, limited insurance coverage has, to a degree, promoted the practice of self-treatment [22-24].

Self-treatment is a self-initiated behaviour [25]. Hence, it is influenced by various sociodemographic, economic, behavioral and health factors [26-29]. Studies on factors related to the choice of self-treatment should be of interest to public health researchers and policymakers involved in planning and development of health care services.

The aims of this study were to: 1) identify the proportion of individuals choosing different treatment options in the Chinese population when experiencing symptoms (no treatment, self-treatment, and professional medical services); 2) identify the factors related to the choice of self-treatment; and 3) identify whether there is a difference between urban and rural residents in their self-treatment decision-making.

## Methods

The data is available from the China National Health Surveys (CNHS), which had been organized and completed by the Ministry of Health of China every fifth year since 1993. The CHNS, which is approved by the National Bureau of Statistics of China, was conducted in 1993 (n = 215,163), 1998 (n = 216,101), 2003(n = 193,689), and 2008 (n = 177,501). A multi-stage stratified random cluster sampling method was used each year to select participants [30-35]. All four surveys were conducted in the same sample areas, whereas all households were randomly selected again. In the first sample stage, all cities or counties in the Mainland of China were stratified based on socio-economic, health care and population structure to sample cities or counties (92 in 1993, 95 in 1998, 95 in 2003, and 94 in 2008). In the second stage, all streets (urban area) or townships (rural area) in the sample cities or counties were stratified based on population size and income per capita to sample streets or townships (460 in 1993, 475 in 1998, 475 in 2003, and 470 in 2008). In the third stage, two residential committees (urban area) or villages (rural area) were selected in each street or township. 60 households were selected from each residential committee or village at random, resulting in 54984 households in 1993, 56000 households in 1998, 57000 households in 2003, and 56400 households in 2008. All members of selected households were invited to participate in the survey. In all four surveys, the CHNS response rate was above 95%.

In the survey, self-treatment was defined as taking some drugs and/or other home remedies, or having a massage and/or physiotherapy rather than visiting a physician when experiencing symptoms or complaints during the two-week period preceding the survey [31]. Self-treatment was assessed by the question: when you had a symptom or complaint during the two-week period preceding the survey, which treatment methods did you implement? The response options were: 1) no treatment; 2) self-treatment; 3) self-treatment and visited physician; 4) visited physician [36]. Differences in proportions were compared using Chi-squared tests and the significance level was set at p < 0.05.

Logistic regression approaches are more convenient than probit or linear probability models for analyzing health care use because the estimated coefficients can easily be converted into odds ratios [37-39].We choose to use a multinomial logit selection model. We have a selection variable, *z*, which takes values 0, 1… *J* for *J* + 1 outcomes. In our case, there are 3 outcomes: no treatment, self-treatment, or professional medical service. The model for determining *z* is

$$P_{\mathrm{ij}}=\text{Prob}\left[Z_{i}=j\right]=\text{exp}\left({\alpha^{\prime }}_{j}\nu_{i}\right)/\left[1+\sum_{j=1}^{J}\text{exp}\left({\alpha^{\prime }}_{j}\nu_{i}\right)\right]$$

where ‘*i*’ is the observation and ‘*j*’ is the choice or outcome. Selection is based on *z*_*i*_ *= j*. *α* is a vector of unknown parameters and *v*_*i*_ is a vector of explanatory variables [40].

We merged the1993, 1998, 2003 and 2008 results databases, and excluded data of participants who had not been ill in the two-week period of interest (n = 118,565, Table 1).

**Table 1:** Number of respondents reporting illness and their treatment choices, by year

**Indicators**	**1993**	**1998**	**2003**	**2008**
Total respondents	215,163	216,101	193,689	177,501
Respondents reporting illness	29565	31244	26602	31154
Urban:				
2-week prevalence (%)	17.5	18.7	15.3	22.2
Doing nothing (%)	9.6	6.2	9.7	6.4
Using self-treatment (%)	31.2	43.5	47.2	31.0
Using self-treatment and professional medical service (%)	0.3	4.7	9.7	32.6^a^
Seeking professional medical service (%)	58.9	45.6	33.3	30.1
Rural:				
2-week prevalence (%)	12.8	13.7	14.0	17.7
Doing nothing (%)	18.3	11.5	14.4	12.5
Using self-treatment (%)	14.9	21.4	31.4	25.3
Using self-treatment and professional medical service (%)	0.3	3.6	8.2	19.1^a^
Seeking professional medical service (%)	66.4	63.5	46.0	43.1

Source: Author’s calculations from the four CNHS and the China Health Statistics Yearbook.

^a^ Patients who had already seen a doctor 2 weeks ago, but continued to treatment.

## Results

The proportion of survey respondents who had opted for self-treatment of their symptom or complaint during the 2-week period preceding the survey was 20% in 1993, 28% in 1998, 36% in 2003 and 27% in 2008 (Table 1). In each year, more urban residents reported self-treating than rural residents (p ≤ 0.01, Table 1).

Table 2 shows that self-treatment behavior differs according to age, profession, and number of family members. There were a significantly larger proportion of respondents opting for self-treatment in the higher age and education categories. Conversely, there were significantly smaller proportions of participants opting for self-treatment in the higher number of family members categories. Significantly greater proportions of working and retired respondents opted for self-treatment compared with respondents who were unemployed or farmers.

**Table 2:** Demographic characteristics of individuals using self-treatment, by year (%)

**Variables**	**1993**	**1998**	**2003**	**2008**	***χ***^**2**^
Gender:					
Male	19.8	28.1	35.5	26.9	2.2
Female	20.3	28.6	35.9	27.2	
*χ*^2^	1.1	1.0	0.4	0.6	
Age:					
15–24 years	15.6	22.1	28.0	20.4	910.0**
25-44 years old	19.9	28.0	33.6	27.0	363.8**
45-64 years old	23.6	31.7	37.9	29.6	162.6**
≥65 years old	26.0	33.1	40.2	26.7	457.5**
*χ*^2^	263.2**	273.5**	211.6**	145.1**	
Marriage:					
Unmarried	21.6	30.4	32.8	28.1	156.0**
Married	21.8	30.3	37.1	27.9	21.9**
Divorced	26.9	32.6	41.1	28.1	5.4
Widowed	24.7	31.5	38.3	28.7	87.0**
*χ*^2^	12.3**	2.1	15.6**	1.3	
Education:					
Illiterate or semi-illiterate	19.2	25.6	32.7	26.7	64.0**
Primary/junior school	21.7	29.5	37.0	28.6	127.5**
High or technical school	30.1	40.7	44.8	28.5	89.8**
College or university and above	31.3	44.5	49.1	27.8	
*χ*^2^	175.1**	359.9**	188.4**	9.5*	
Profession:					
Unemployed	17.4	32.6	43.0	29.9	793.7**
Worker	28.8	42.2	44.3	30.0	2400.0**
Farmer	15.2	21.5	31.3	26.3	1000.0**
Retired	34.6	38.8	49.6	26.0	1500.0**
*χ*^2^	796.0**	1000.0**	572.5**	49.6**	
Family members:					
1–2 persons	24.1	33.2	40.8	28.5	207.1**
3-4 persons	21.7	30.9	38.0	27.7	312.7**
≥5 persons	17.5	23.4	31.7	24.5	581.9**
*χ*^2^	86.8**	211.5**	120.4**	31.5**	

*Significant at 5%. **Significant at 1%.

Table 3 shows the proportion of respondents within different economic categories who opted for self-treatment. In 1993, 1998, and 2003 the lowest proportions of respondents opting for self-treatment were among those who were without health insurance, who had other medical insurance, or who were in a rural cooperative medical scheme. In 2008, the lowest proportion of respondents opting for self-treatment was among those with free medical insurance. The proportion opting for self-treatment was significantly lower among those with no sanitary water than those with sanitary water, and in 1998 and 2003, among those with no TV (compared with those with a TV). Significantly higher proportions of respondents living in poverty opted for self-treatment in 1998 and 2008 (compared with those not living in poverty), but the inverse was true in 2003.

**Table 3:** The proportion of respondents using self-treatment in different economic categories, by year (%)

**Variables**	**1993**	**1998**	**2003**	**2008**	***χ***^**2**^
Insurance:					
Without any health insurance	16.0	25.3	33.9	35.9	7200.0**
Other medical insurance	12.9	24.6	32.0	32.4	251.7**
Rural cooperative medical scheme	15.0	18.5	29.0	25.4	13000.0**
Urban basic medical insurance	33.4	44.3	47.7	27.6	301.6**
Free medical insurance	30.5	41.9	49.5	22.8	1300.0**
*χ*^2^	1000.0**	907.9**	367.2**	191.7**	
Shortest distances from residence to medical institutions:					
≤1 km	21.0	29.7	38.1	27.3	275.7**
>1 km	17.8	25.1	31.0	26.6	
*χ*^2^	38.2**	66.3**	135.2**	2.2	
Annual per capita income^a^:					
Low	17.5	21.9	30.6	23.8	2900.0**
Middle	19.9	30.3	37.9	27.1	241.2**
High	4.5	39.3	44.2	27.7	3300.0**
*χ*^2^	15.3**	655.1**	350.0**	22.4**	
TV:					
No	NA	21.3	29.0	27.2	340.9**
Yes	NA	29.7	36.9	27.1	
*χ*^2^		148.7**	90.7**	0.01	
Sanitary water:					
No	14.3	20.6	29.5	24.6	27.5**
Yes	27.7	36.7	41.8	29.0	
*χ*^2^	833.9**	1000.0**	460.9**	80.0**	
Family poverty:					
No	NA	28.2	36.0	26.9	191.4**
Yes	NA	31.3	31.2	28.6	
*χ*^2^		10.6**	15.2**	4.5*	

^a^ Annual per capita income discounted by CPI inflation in 1993, 1998 and 2003, and divided into low, middle, and high tertiles. NA: no data available.

*Significant at 5%. **Significant at 1%.

Table 4 shows the proportion of participants within each health behavior category who opted for self-treatment, by year. In 1993 and 2003, a significantly greater proportion of respondents who smoked opted for self-treatment (compared with those who did not smoke). In 1993 and 2008, a significantly greater proportion of respondents who drank alcohol opted for self-treatment (compared with those who did not drink alcohol). In all years, a significantly greater proportion of respondents who exercised opted for self-treatment (compared with those who did not exercise). Daily health status was assessed by the presence of chronic illness and/or limited activity. In all years, a significantly greater proportion of respondents with a chronic illness than without opted for self-treatment. In 2003 and 2008, a significantly smaller proportion of respondents with limited activity than without opted for self-treatment.

**Table 4:** The proportion of respondents using self-treatment in different health-behavior and daily health status categories, by year (%)

**Variables**	**1993**	**1998**	**2003**	**2008**	***χ***^**2**^
Smoking:					
No	19.1	30.1	36.7	28.1	2000.0**
Yes	23.2	31.3	38.2	26.1	
*χ*^2^	54.7**	3.5	4.5*	2.3	
Drinking:					
No	19.6	30.3	36.9	27.7	192.0**
Yes	23.5	31.2	38.7	31.5	
*χ*^2^	31.8**	1.1	2.6	21.8**	
Exercising:					
No	18.8	28.1	34.8	27.7	215.6**
Yes	29.5	40.8	47.0	29.3	
*χ*^2^	223.9**	300.7**	224.1**	7.5**	
Chronic illness:					
No	17.2	25.6	31.5	25.4	466.8**
Yes	24.2	31.6	39.7	28.1	
*χ*^2^	224.5**	143.4**	201.7**	27.3**	
Limited activity limiter:					
No	20.0	28.5	37.9	28.7	1400.0**
Yes	20.4	27.1	30.2	25.3	
*χ*^2^	0.3	1.5	147.7**	24.3**	

*Significant at 5%. **Significant at 1%.

Table 5 shows the proportion of participants within each illness symptom, severity, duration and course category, by year. There were significant differences in the proportion opting for self-treatment each year according to illness symptom, severity and duration (both p ≤ 0.01). In terms of course of illness, a significantly greater proportion of chronically ill patients opted for self-treatment, relative to the other categories (p ≤ 0.01).

**Table 5:** The proportion of respondents using self-treatment in different self-perceived illness status categories, by year (%)

**Variables**	**1993**	**1998**	**2003**	**2008**	***χ***^**2**^
Illness symptoms:					
Pain	NA	28.2	35.1	29.1	34.0**
Fever	NA	21.4	25.8	20.8	831.9**
Palpitation	NA	28.0	38.4	26.2	45.3**
Other	NA	33.2	37.5	27.1	298.6**
*χ*^2^		328.3**	141.0**	83.6**	
Illness severity:					
Not serious	NA	34.9	42.5	32.8	148.3**
General	NA	29.3	38.0	27.5	114.3**
Serious	NA	19.5	24.8	20.1	10.7**
*χ*^2^		459.2**	497.8**	285.5**	
Duration of illness (within 2 weeks):					
1-2 days	24.9	34.6	37.9	31.4	48.9**
3-4 days	19.0	28.2	35.3	27.1	28.1**
≥5 days	18.1	25.7	34.7	24.9	12.7**
*χ*^2^	148.9**	190.7**(0.000)	21.2**	108.6**	
Course of illness:					
Acute illness occurred within 2 weeks	NA	27.4	33.3	26.6	773.6**
Acute illness occurred 2 weeks prior	NA	19.6	23.5	17.3	5.2
chronic illness	NA	31.5	39.6	28.4	804.8**
*χ*^2^		162.9**	257.3**	133.7**	

Source: Author’s calculations from the four CNHS.

NA: no data available.

*Significant at 5%. **Significant at 1%.

Table 6 shows the final results of the logit model for the individual’s choice of seeking professional medical services, using self-treatment, or doing nothing (the reference). Variables missing data for a survey year (TV, family poverty, illness symptom, illness severity, and illness course) and those found non-significant in the above chi-square analyses (gender, marriage) were not included in the model. Most variables and dummy variables in the model for explaining the probability of using self-treatment, and the probability of seeking professional services, were significant (p ≤ 0.05). In general, the explanatory variables had the expected signs.

**Table 6:** Odds ratios and 95% confidence intervals (CI) of a multinomial logit model for the choice of using self-treatment, seeking professional medical services, or doing nothing (reference)

**Variables**	**Prob(Y) = Self-treatment**	**Prob(Y) = Professional medical service**
Survey year:1993 (reference)		
1998	2.19 (1.84–2.62)**	0.87(0.75-1.01)
2003	2.37(1.98-2.83)**	0.54(0.46-0.62)**
2008	1.98(1.64-2.39)**	0.49(0.41-0.58)**
Urban:Rural (reference group)	1.32(1.21-1.44)**	0.90(0.83-0.98)*
Age: 15–24 years old (=reference group)		
25-44 years old	0.92(0.82-1.03)	0.83(0.74-0.93)**
45-64 years old	0.99(0.88-1.12)	0.74(0.66-0.83)**
>65 years old	1.01(0.89-1.15)	0.80(0.71-0.90)**
Education: Illiterate or semi-illiterate(=reference group)		
Primary or junior school	1.17(1.10-1.24)**	1.09(1.03-1.15)**
High or technical school	1.20(1.08-1.33)**	1.06(0.97-1.18)
College or university and above	1.09(0.92-1.27)	1.03(0.88-1.21)
Profession: Unemployed (=reference group)		
Worker	1.22(1.10-1.35)**	1.27(1.15-1.40)**
Farmer	0.85(0.79-0.92)**	1.15(1.07-1.24)**
Retired	1.13(0.99-1.29)	1.28(1.12-1.47)**
Family members: 1–2 person (=reference group)		
3-4 persons	1.29(1.16-1.45)**	1.24(1.12-1.39)**
≥5 persons	1.39(1.24-1.56)**	1.57(1.41-1.76)**
Insurance: Without insurance(=reference group)		
Other medical insurance	0.97(0.82-1.15)	1.36(1.15-1.61)**
Rural cooperative medical scheme	0.87(0.80-0.94)**	1.05(0.98-1.14)
Urban basic medical insurance	1.03(0.93-1.15)	1.25(1.12-1.40)**
Free medical insurance	1.45(1.22-1.72)**	1.65(1.39-1.95)**
Shortest distances from residence at medical institutions:≤1 km (=reference group)	0.83(0.79-0.88)**	0.82(0.78-0.86)**
Sanitary water: No (=reference group)	1.15(1.08-1.22)**	0.86(0.82-0.91)**
Annual per capita income^a^: Low(=reference group)		
Middle	1.53(1.44-1.63)**	1.46(1.38-1.55)**
High	1.59(1.47-1.72)**	1.72(1.59-1.85)**
Smoking:No (=reference group)	1.01(0.95-1.08)	0.91(0.86-0.97)**
Drinking:No (=reference group)	0.92(0.86-1.0)*	0.84(0.78-0.90)**
Exercising: No (=reference group)	1.23(1.14-1.33)**	1.22(1.13-1.32)**
Limited activity: No (=reference group)	0.84(0.78-0.90)**	0.96(0.90-1.13)**
Chronic disease: No (=reference group)	1.21(1.14-1.27)**	0.86(0.82-0.91)**
Duration within two week:1-2 day (=reference group)		
3-4 day	1.32(1.24-1.41)**	1.87(1.76-2.0)**
≥5 day	1.0 (0.94-1.07)	1.62(1.52-1.72)**
Constant	−0.68(0.12)**	1.03(0.11)**

Number of observations = 69,815; Log likelihood = −65984.0; LR-*χ*^2^ = 5707.86, P > *χ*^2^ = 0.000.

*Significant at 5%. **Significant at 1%. Constant is a coefficient.

Using 1993 as the reference, the survey year had a positive association with the probability of self-treating, and a negative association with the probability of seeking professional medical services. Compared with rural respondents, urban respondents had a higher probability of self-treating, and a lower probability of seeking professional medical services. Age had a negative association with the probability of seeking professional medical services. Compared with respondents who were illiterate or semi-illiterate, those with up to technical school education had significantly higher odds of self-treating, although those with primary or junior school education were also more likely to seek professional medical services. Compared with unemployed respondents, workers were more likely to self-treat, but the inverse was true for farmers. On the other hand, compared with the unemployed, all other professional categories were more likely to seek professional medical services. The farmers’ probability of seeking professional medical services was higher than that of the unemployed group, but lower than that of the other professional groups. Using 1–2 persons as the reference group, when family members increased, tendency to self-treatment or professional medical services increased. Compared with respondents without insurance, those with free medical insurance were more likely to use self-treatment, but the inverse was true for those in the rural cooperative medical scheme. Those with other, urban basic or free medical insurance were more likely than those with no insurance to seek professional medical services. Compared with respondents living > 1 km from a medical institution, those who lived ≤ 1 km away were more likely to self-treat or seek medical services. Those with middle or high annual incomes were more likely than those with low incomes to use self-treatment or seek professional medical services. Respondents with sanitary water or a chronic disease also had a greater probability of using self-treatment, and a lower probability of seeking professional medical services compared with those with no sanitary water or chronic disease. Respondents who exercised had higher probabilities of both using self-treatment and seeking professional medical services relative to their counterparts, but the inverse was true for respondents who drank alcohol or had limited activity. Smokers had a significantly lower probability of seeking professional medical services than non-smokers. Compared with short duration illnesses (1–2 days), those with illnesses of 3–4 days duration had higher probabilities of both using self-treatment and seeking professional medical services, while those with illnesses for ≥ 5 days had a higher probability of seeking professional medical services only.

## Discussion

In developing countries, where an individual’s financial resources are often scarce, health care utilization is not always the highest priority, and even when ill an individual may choose not to seek health care [40]. Without taking economic and social conditions into account there are three main health care scenarios: First, if there is a surplus of professional medical services compared with medical needs, the growth rate of professional medical services use should be faster than that of self-treatment. Second, if there is a relative supply and demand balance, the growth rate of both will remain at a steady level. Third, if there is a gap in professional medical services, the self-treatment growth rate may be faster. According to our findings, China fits into the third scenario. We can conclude that China's self-treatment and professional medical services have shared the incremental medical needs of residents in recent years. Should all those that use self-treatment shift to using professional medical services, the medical institutions would not be able to cope. Therefore, at this stage self-treatment should be an important supplemental channel to professional medical services.

Our study revealed that the prevalence of self-treatment with a recall period of two-weeks significantly differed in urban and rural population (31.2% vs 14.9% in 1993, 43.5% vs 21.4% in 1998, 47.2% vs 31.4% in 2003, 31.0% vs 25.3% in 2008) in China. Similar results were reported by other studies. A study in Portugal reported an urban (26.2%) - rural (21.5%) difference in self-treatment [41,42]. In India, self-treatment prevalence was 37% in urban and 17% in rural population [43]. It is difficult to compare with other studies in the prevalence of self-treatment due to the use of different definitions of self-treatment.

In the analysis of individual health care decision-making, we found that self-perceived illness status, economic condition, and individual health-behavior were important factors. These individual factors construct an internal-dynamic mechanism of self-treatment selection. The effect of different illness symptoms (severity, duration) on the probability to self-treat shows a certain degree of rationality. However, educational and economic variables also seem to have persuasive effects on health care choices. With increasing education and income the probability of self-treatment also increases. Some scholars consider that self-treatment is an economic-restraint phenomenon [16], and that the proportion of highly educated individuals who use self-treatment should decrease because they may have gained a higher income than others. The profession, TV, and sanitary water associations we found support this hypothesis. This finding of an economic relationship with the use of self-treatment is an important consideration when rethinking health care in China. Gender and marriage had no impact on the decision to self-treat, in accordance with Hjortsberg’s study [40].

Drug accessibility is also an important determinant of the use of self-treatment. Pharmacies are included in the CNHS in the assessment of distances from medical institutions. In recent years, the accessibility of pharmacies has substantially increased in rural and urban areas because the Chinese Government has implemented the construction of a drug sales network [44].We found that living in an urban area increased the likelihood to self-treat. This may be because urban infrastructure is superior to that of rural areas. The use of self-treatment is relative to price and time costs. It has been found that urban residents are likely to choose cheap, easy self-treatments when their illness symptoms are minor, common and just beginning [11]. The switch of prescription to OTC drugs has been deregulated in China, and like as Hubertus Cranz, the Association of the European Self-Medication Industry (AESGP) director general said, taking into account the growing recognition of the economic and public health value of self-medication, many opportunities lie ahead of us [45].

Insurance is a system factor related to the use of self-treatment. Most other medical insurance and rural cooperative medical schemes have been based on inpatient or serious-illness accounts; but urban basic medical insurance and free medical insurance have been based on both outpatient and inpatient accounts. Those who have the former insurance will not be reimbursed for the drugs acquired as an outpatient, while those who have the latter insurance can be reimbursed for such drugs once every quarter or year [22,46]. Therefore, insurance status has a significant impact on the choice of using public village clinics relative to self-treatment [47]. At present, China is implementing the New Cooperative Medical Scheme (NCMS) and urban basic medical insurance system to achieve universal medical insurance coverage. We believe that this will have a significant impact on self-treatment.

There are two major limitations to this study. A major limitation is that cross-sectional study does not establish cause-effect relationship between factors and self-treatment, even after controlling for potential confounders. A second limitation is that it is difficult to compare with other studies due to the inconsistencies of definitions of self-treatment. Owing to time constraints, we did not conduct in-depth interviews with self-treatment stakeholders regarding its development. Meanwhile, relevant research in China is lacking, especially analyses of the CNHS data. Therefore, it is difficult to provide a context for our results. This study is only a preliminary characterization of self-treatment in China. The next step is to focus on self-treatment among the elderly, women and children, and those with chronic diseases.

## Conclusions

In China, self-medication and professional medical services have shared the incremental medical needs of residents in recent years. Compared with rural area, living in urban area increased the likelihood of self-treatment, and decreased the likelihood to seek professional medical services. The decision to self-treat is complex, and involves an interaction between internal-dynamic and external-strengthening mechanisms, including individual (age, education, profession, health-behaviors, and illness status), household (family members, TV, and sanitary water), accessibility and medical insurance system factors. In particular, we found that self-perceived illness status, economic circumstances, and education play important roles. Gender or marriage had no impact on the decision to self-treat. These findings provide information for us to consider when rethinking and developing strategies related to self-treatment in China.

## Competing interests

The authors declare that they have no competing interests.

## Authors’ contributions

YFL: designed the study, took part in the statistical analysis and drafted the manuscript. KQR and XWR assisted with the data analysis and reviewed the manuscript. All authors read and approved the final manuscript.

## Pre-publication history

The pre-publication history for this paper can be accessed here:

http://www.biomedcentral.com/1471-2458/12/995/prepub
